# Supplementary material for: Collection of economic data using UB-04s: Is it worth the effort? Evidence from two clinical trials
Source: PLoS One. 2022 Nov 17;17(11):e0277685. doi: 10.1371/journal.pone.0277685 (PMC9671305; doi:10.1371/journal.pone.0277685)
Supplement: S1 Fig — Source: https://www.cdc.gov/wtc/pdfs/policies/ub-40-P.pdf. (PDF) [file pone.0277685.s001.pdf]

1	2	3a PAT. CNTL #		4 TYPE OF BILL
		b. MED. REC. #		
		5 FED. TAX NO.	6 STATEMENT COVERS PERIOD FROM / THROUGH	7

8 PATIENT NAME	a	9 PATIENT ADDRESS	a
b		b	c	d	e

10 BIRTHDATE	11 SEX	ADMISSION 12 DATE	13 HR	14 TYPE	15 SRC	16 DHR	17 STAT	CONDITION CODES 18	19	20	21	22	23	24	25	26	27	28	29 ACDT STATE	30

	31 OCCURRENCE CODE	DATE	32 OCCURRENCE CODE	DATE	33 OCCURRENCE CODE	DATE	34 OCCURRENCE CODE	DATE	35 OCCURRENCE SPAN CODE	FROM	THROUGH	36 OCCURRENCE SPAN CODE	FROM	THROUGH	37	
a																a
b																b

38		39 VALUE CODES CODE	AMOUNT	40 VALUE CODES CODE	AMOUNT	41 VALUE CODES CODE	AMOUNT
	a						
	b						
	c						
	d						

	42 REV. CD.	43 DESCRIPTION	44 HCPCS / RATE / HIPPS CODE	45 SERV. DATE	46 SERV. UNITS	47 TOTAL CHARGES	48 NON-COVERED CHARGES	49	
1									1
2									2
3									3
4									4
5									5
6									6
7									7
8									8
9									9
10									10
11									11
12									12
13									13
14									14
15									15
16									16
17									17
18									18
19									19
20									20
21									21
22									22
23		***PAGE ____ OF ____***	***CREATION DATE***		***TOTALS***				23

	50 PAYER NAME	51 HEALTH PLAN ID	52 REL. INFO	53 ASG. BEN.	54 PRIOR PAYMENTS	55 EST. AMOUNT DUE	56 NPI		
A							57		A
B							OTHER		B
C							PRV ID		C

	58 INSURED'S NAME	59 P. REL	60 INSURED'S UNIQUE ID	61 GROUP NAME	62 INSURANCE GROUP NO.	
A						A
B						B
C						C

	63 TREATMENT AUTHORIZATION CODES	64 DOCUMENT CONTROL NUMBER	65 EMPLOYER NAME	
A				A
B				B
C				C

66 DX	67	A	B	C	D	E	F	G	H	68
	I	J	K	L	M	N	O	P	Q	

69 ADMIT DX		70 PATIENT REASON DX	a	b	c	71 PPS CODE		72 ECI	a	b	c	73

74 PRINCIPAL PROCEDURE CODE / DATE	a. OTHER PROCEDURE CODE / DATE	b. OTHER PROCEDURE CODE / DATE	75
c. OTHER PROCEDURE CODE / DATE	d. OTHER PROCEDURE CODE / DATE	e. OTHER PROCEDURE CODE / DATE	

76 ATTENDING	NPI	QUAL	
LAST		FIRST	
77 OPERATING	NPI	QUAL	
LAST		FIRST	
78 OTHER	NPI	QUAL	
LAST		FIRST	
79 OTHER	NPI	QUAL	
LAST		FIRST	

80 REMARKS	81CC a		
	b		
	c		
	d		

UB-04 NOTICE: THE SUBMITTER OF THIS FORM UNDERSTANDS THAT MISREPRESENTATION OR FALSIFICATION OF ESSENTIAL INFORMATION AS REQUESTED BY THIS FORM, MAY SERVE AS THE BASIS FOR CIVIL MONETARTY PENALTIES AND ASSESSMENTS AND MAY UPON CONVICTION INCLUDE FINES AND/OR IMPRISONMENT UNDER FEDERAL AND/OR STATE LAW(S).

Submission of this claim constitutes certification that the billing information as shown on the face hereof is true, accurate and complete. That the submitter did not knowingly or recklessly disregard or misrepresent or conceal material facts. The following certifications or verifications apply where pertinent to this Bill:

1. If third party benefits are indicated, the appropriate assignments by the insured /beneficiary and signature of the patient or parent or a legal guardian covering authorization to release information are on file. Determinations as to the release of medical and financial information should be guided by the patient or the patient's legal representative.
2. If patient occupied a private room or required private nursing for medical necessity, any required certifications are on file.
3. Physician's certifications and re-certifications, if required by contract or Federal regulations, are on file.
4. For Religious Non-Medical facilities, verifications and if necessary re-certifications of the patient's need for services are on file.
5. Signature of patient or his representative on certifications, authorization to release information, and payment request, as required by Federal Law and Regulations (42 USC 1935f, 42 CFR 424.36, 10 USC 1071 through 1086, 32 CFR 199) and any other applicable contract regulations, is on file.
6. The provider of care submitter acknowledges that the bill is in conformance with the Civil Rights Act of 1964 as amended. Records adequately describing services will be maintained and necessary information will be furnished to such governmental agencies as required by applicable law.
7. For Medicare Purposes: If the patient has indicated that other health insurance or a state medical assistance agency will pay part of his/her medical expenses and he/she wants information about his/her claim released to them upon request, necessary authorization is on file. The patient's signature on the provider's request to bill Medicare medical and non-medical information, including employment status, and whether the person has employer group health insurance which is responsible to pay for the services for which this Medicare claim is made.
8. For Medicaid purposes: The submitter understands that because payment and satisfaction of this claim will be from Federal and State funds, any false statements, documents, or concealment of a material fact are subject to prosecution under applicable Federal or State Laws.
9. For TRICARE Purposes:

   (a) The information on the face of this claim is true, accurate and complete to the best of the submitter's knowledge and belief, and services were medically necessary and appropriate for the health of the patient;

   (b) The patient has represented that by a reported residential address outside a military medical treatment facility catchment area he or she does not live within the catchment area of a U.S. military medical treatment facility, or if the patient resides within a catchment area of such a facility, a copy of Non-Availability Statement (DD Form 1251) is on file, or the physician has certified to a medical emergency in any instance where a copy of a Non-Availability Statement is not on file;

   (c) The patient or the patient's parent or guardian has responded directly to the provider's request to identify all health insurance coverage, and that all such coverage is identified on the face of the claim except that coverage which is exclusively supplemental payments to TRICARE-determined benefits;

   (d) The amount billed to TRICARE has been billed after all such coverage have been billed and paid excluding Medicaid, and the amount billed to TRICARE is that remaining claimed against TRICARE benefits;

   (e) The beneficiary's cost share has not been waived by consent or failure to exercise generally accepted billing and collection efforts; and,

   (f) Any hospital-based physician under contract, the cost of whose services are allocated in the charges included in this bill, is not an employee or member of the Uniformed Services. For purposes of this certification, an employee of the Uniformed Services is an employee, appointed in civil service (refer to 5 USC 2105), including part-time or intermittent employees, but excluding contract surgeons or other personal service contracts. Similarly, member of the Uniformed Services does not apply to reserve members of the Uniformed Services not on active duty.

   (g) Based on 42 United States Code 1395cc(a)(1)(j) all providers participating in Medicare must also participate in TRICARE for inpatient hospital services provided pursuant to admissions to hospitals occurring on or after January 1, 1987; and

   (h) If TRICARE benefits are to be paid in a participating status, the submitter of this claim agrees to submit this claim to the appropriate TRICARE claims processor. The provider of care submitter also agrees to accept the TRICARE determined reasonable charge as the total charge for the medical services or supplies listed on the claim form. The provider of care will accept the TRICARE-determined reasonable charge even if it is less than the billed amount, and also agrees to accept the amount paid by TRICARE combined with the cost-share amount and deductible amount, if any, paid by or on behalf of the patient as full payment for the listed medical services or supplies. The provider of care submitter will not attempt to collect from the patient (or his or her parent or guardian) amounts over the TRICARE determined reasonable charge. TRICARE will make any benefits payable directly to the provider of care, if the provider of care is a participating provider.

Source: https://www.cdc.gov/wtc/pdfs/policies/ub-40-P.pdf
