# Supplementary material for: Collection of economic data using UB-04s: Is it worth the effort? Evidence from two clinical trials
Source: PLoS One. 2022 Nov 17;17(11):e0277685. doi: 10.1371/journal.pone.0277685 (PMC9671305; doi:10.1371/journal.pone.0277685)
Supplement: Attachment — Submitted filename: Response letter.docx [file pone.0277685.s004.docx]

Dear Editor,

Thank you for the invitation to revise and resubmit our manuscript “Collection of economic data using UB-04s: is it worth the effort? Evidence from two clinical trials” (PONE-D-21-30170) for publication at PLOS ONE.

We have answered to the editor and reviewer comments; the detailed answers can be found below:

Journal Requirements:

When submitting your revision, we need you to address these additional requirements.

1. Please ensure that your manuscript meets PLOS ONE's style requirements, including those for file naming. The PLOS ONE style templates can be found at 
[https://journals.plos.org/plosone/s/file?id=wjVg/PLOSOne_formatting_sample_main_body.pdf](https://urldefense.com/v3/__https:/journals.plos.org/plosone/s/file?id=wjVg*PLOSOne_formatting_sample_main_body.pdf__;Lw!!NFcUtLLUcw!X8MNFLtBlSgg1grTEzuk3Xy5PjrJKTVfO9lp2ydgIYrm27aS1i9_aQBgXqr26HUa3hflqzgsrhExTwZaN4yd7JE$) and 
[https://journals.plos.org/plosone/s/file?id=ba62/PLOSOne_formatting_sample_title_authors_affiliations.pdf](https://urldefense.com/v3/__https:/journals.plos.org/plosone/s/file?id=ba62*PLOSOne_formatting_sample_title_authors_affiliations.pdf__;Lw!!NFcUtLLUcw!X8MNFLtBlSgg1grTEzuk3Xy5PjrJKTVfO9lp2ydgIYrm27aS1i9_aQBgXqr26HUa3hflqzgsrhExTwZa09mrDHc$)

**ANS: we have formatted the manuscript to follow PLOS ONE style requirements.**

2. Thank you for stating the following in the Financial Disclosure section: "LH, EI, JH, and RH are all employees of Medtronic plc. XL is a former employee of Medtronic plc."

We note that you received funding from a commercial source: [Name of Company]
Please provide an amended Competing Interests Statement that explicitly states this commercial funder, along with any other relevant declarations relating to employment, consultancy, patents, products in development, marketed products, etc.

Within this Competing Interests Statement, please confirm that this does not alter your adherence to all PLOS ONE policies on sharing data and materials by including the following statement: "This does not alter our adherence to PLOS ONE policies on sharing data and materials.” (as detailed online in our guide for authors [http://journals.plos.org/plosone/s/competing-interests).](https://urldefense.com/v3/__http:/journals.plos.org/plosone/s/competing-interests).__;!!NFcUtLLUcw!X8MNFLtBlSgg1grTEzuk3Xy5PjrJKTVfO9lp2ydgIYrm27aS1i9_aQBgXqr26HUa3hflqzgsrhExTwZa5wDKXew$)  If there are restrictions on sharing of data and/or materials, please state these. Please note that we cannot proceed with consideration of your article until this information has been declared.

Please include your amended Competing Interests Statement within your cover letter. We will change the online submission form on your behalf.

**ANS: we have updated our cover letter, adding a statement on the funding of the commercial source (Medtronic) and stating the role of the funder in the study. The following statement regarding funding and competing interests was added: "This study was funded by Medtronic, as well as the clinical trials used in it; this does not alter our adherence to PLOS ONE policies on sharing data and materials. The publication of study results was not contingent on Medtronic’s approval or censorship of the manuscript. All authors, except for Dr. Lu, currently receive wages and benefits from Medtronic, and are minority stockholders of Medtronic. Dr. Lu received wages and benefits from Medtronic as a former employee."**

3. In your Data Availability statement, you have not specified where the minimal data set underlying the results described in your manuscript can be found. PLOS defines a study's minimal data set as the underlying data used to reach the conclusions drawn in the manuscript and any additional data required to replicate the reported study findings in their entirety. All PLOS journals require that the minimal data set be made fully available. For more information about our data policy, please see [http://journals.plos.org/plosone/s/data-availability](https://urldefense.com/v3/__http:/journals.plos.org/plosone/s/data-availability__;!!NFcUtLLUcw!X8MNFLtBlSgg1grTEzuk3Xy5PjrJKTVfO9lp2ydgIYrm27aS1i9_aQBgXqr26HUa3hflqzgsrhExTwZaUjbEMoY$).

Upon re-submitting your revised manuscript, please upload your study’s minimal underlying data set as either Supporting Information files or to a stable, public repository and include the relevant URLs, DOIs, or accession numbers within your revised cover letter. For a list of acceptable repositories, please see [http://journals.plos.org/plosone/s/data-availability#loc-recommended-repositories](https://urldefense.com/v3/__http:/journals.plos.org/plosone/s/data-availability*loc-recommended-repositories__;Iw!!NFcUtLLUcw!X8MNFLtBlSgg1grTEzuk3Xy5PjrJKTVfO9lp2ydgIYrm27aS1i9_aQBgXqr26HUa3hflqzgsrhExTwZa6KBaXk4$). Any potentially identifying patient information must be fully anonymized.

Important: If there are ethical or legal restrictions to sharing your data publicly, please explain these restrictions in detail. Please see our guidelines for more information on what we consider unacceptable restrictions to publicly sharing data: [http://journals.plos.org/plosone/s/data-availability#loc-unacceptable-data-access-restrictions](https://urldefense.com/v3/__http:/journals.plos.org/plosone/s/data-availability*loc-unacceptable-data-access-restrictions__;Iw!!NFcUtLLUcw!X8MNFLtBlSgg1grTEzuk3Xy5PjrJKTVfO9lp2ydgIYrm27aS1i9_aQBgXqr26HUa3hflqzgsrhExTwZaXG3JflM$). Note that it is not acceptable for the authors to be the sole named individuals responsible for ensuring data access.

We will update your Data Availability statement to reflect the information you provide in your cover letter.

**ANS: we have provided minimal underlying data sets (3, one per cost study) in the submitted file 'Datasets.zip'.**

4. Please review your reference list to ensure that it is complete and correct. If you have cited papers that have been retracted, please include the rationale for doing so in the manuscript text, or remove these references and replace them with relevant current references. Any changes to the reference list should be mentioned in the rebuttal letter that accompanies your revised manuscript. If you need to cite a retracted article, indicate the article’s retracted status in the References list and also include a citation and full reference for the retraction notice.

[Note: HTML markup is below. Please do not edit.]

Reviewers' comments:

Reviewer's Responses to Questions

**Comments to the Author**

1. Is the manuscript technically sound, and do the data support the conclusions?

The manuscript must describe a technically sound piece of scientific research with data that supports the conclusions. Experiments must have been conducted rigorously, with appropriate controls, replication, and sample sizes. The conclusions must be drawn appropriately based on the data presented.

Reviewer #1: Yes

2. Has the statistical analysis been performed appropriately and rigorously?

Reviewer #1: Yes

3. Have the authors made all data underlying the findings in their manuscript fully available?

The [PLOS Data policy](https://urldefense.com/v3/__http:/www.plosone.org/static/policies.action*sharing__;Iw!!NFcUtLLUcw!X8MNFLtBlSgg1grTEzuk3Xy5PjrJKTVfO9lp2ydgIYrm27aS1i9_aQBgXqr26HUa3hflqzgsrhExTwZaZMhumSg$) requires authors to make all data underlying the findings described in their manuscript fully available without restriction, with rare exception (please refer to the Data Availability Statement in the manuscript PDF file). The data should be provided as part of the manuscript or its supporting information, or deposited to a public repository. For example, in addition to summary statistics, the data points behind means, medians and variance measures should be available. If there are restrictions on publicly sharing data—e.g. participant privacy or use of data from a third party—those must be specified.

Reviewer #1: Yes

4. Is the manuscript presented in an intelligible fashion and written in standard English?

PLOS ONE does not copyedit accepted manuscripts, so the language in submitted articles must be clear, correct, and unambiguous. Any typographical or grammatical errors should be corrected at revision, so please note any specific errors here.

Reviewer #1: Yes

5. Review Comments to the Author

Please use the space provided to explain your answers to the questions above. You may also include additional comments for the author, including concerns about dual publication, research ethics, or publication ethics. (Please upload your review as an attachment if it exceeds 20,000 characters)

Reviewer #1: The current study compares a broad, charge-based costing methodology to a more detailed healthcare resource utilization (HCU) data assignment-based costing methodology across three different healthcare encounter types common to two Medtronic trials with available UB-04 (Medicare summary billing) data. Encounters for 23 CIED infections within the WRAP-IT trial, 66 IDE-related adverse events from the Micra IDE trial, and 108 Micra IDE implant encounters from the same trial were costed using (a) UB-04 total charges converted to costs using publicly available CCRs at a hospital-and-year level and (b) provider costs from Premier Health Data for encounters matched >=2:1 to those in the trials, with matching criteria dependent on the encounter type and place of service, among other variables. Differences in the estimated costs by costing methodology were tested statistically for difference from 0. The authors found that methodologies derived statistically similar costs for the adverse event and infection encounters. Costing methodologies led to statistically different costs among the implant encounters, and neither methodology was considered clearly superior: UB-04s underrepresented true costs on average, and the cost assignment strategy relied on matching trial cases to PHD claims generated one year after the Micra IDE was approved for use, when adoption was still relatively low. The authors appropriately conclude that their total charge UB-04-based and cost-assignment-based costing may both be inadequate in deriving costs for novel interventions without established coding and reimbursement strategies.

In order to guide decisions on whether to invest resources into collection of primary costing data alongside clinical trials, the investigators leveraged their findings to develop a decision tree for selecting costing strategies depending on purpose of the analysis (economic value or reimbursement strategy). The research article adds to the health economics methodological literature, provides a tangible resource for making important cost data collection decisions, and satisfies all criteria for publication in PLOS ONE. I recommend the article for publication after minor revisions.

Study methodology was transparent and succinctly described. The data sources for the different costing strategies are clearly explained, as are the statistical methods used to compare total encounter costs by strategy. Use of GLM modeling with gamma family is appropriate for cost comparisons, and inclusion of patient and site-patient random effects estimates reflects statistical rigor. No mention of the small sample size (23 bills in the WRAP-IT CIED infection comparison, in particular) is made in the limitations or the statistical methodology sections and should be considered.

**ANS: We added a sentence on this study limitation to the discussion section: “And lastly, the sample sizes are relatively small, particularly the sample of the WRAP-IT CIED infection cost analysis, which makes the results susceptible to outliers and parametric choices.”**

There are a few additional minor issues the authors are suggested to address. (1) The paragraph beginning at line 219 is confusing. The authors are suggested to clarify that the total cost estimates that fell below the expected purchase price of the Micra device (line 220) are the charge-based costs rather than the assignment-based costs. This only became clear once it was mentioned in the Discussion section later. (2) Inflation of the costs to 2017 USD for the WRAP-IT infection data and to 2018 USD for the Micra data is unlikely to affect comparability of the cost differences between costing methodologies across the 3 encounter types, but it is unclear why the WRAP-IT cost data are not similarly inflated to 2018 to pointedly ensure this comparability, since total cost comparisons are differences rather than ratios. (3) Line 71-73 regarding the ISPOR Task Force Report on criteria for collecting economic data lacks information. Why are the available resources inadequate for decision making, and/or how will the original research study add important information to the resources for deciding whether to collect UB-04s or claims/administrative data alongside clinical trials? (4) An extra word in line 158 should be removed ("Since health care costs data {are} usually follow..."). (5) A typo in line 248 should be corrected ("These types of HCU could be relevant for procedures {were} outpatient HCUs are frequently used" -- "were" should be "where").

**ANS: Thank you for your comments. Here are our detailed answers:**

**(1) We have clarified that indeed we refer to the charge-based costs being above the price of a Mira device.**

**(2) We adjusted costs in the WRAP-IT CIED infection analysis to 2017 US dollars instead of 2018 US dollars to be consistent with previous economic analysis of the WRAP-IT trial (Wilkoff B et al., 2020). As the reviewer noted, the year chosen to adjust costs does not affect comparability within each cost study. We have added the reason why we adjusted WRAP-IT CIED infection costs to 2017 US dollars instead of 2018 US dollars.**

**(3) The reviewer is correct that the reference to the ISPOR report lacks information. We have added a sentence stating what the report recommends, and what does it lack that this study provides: “This report states the possible trade-offs across accuracy, feasibility, generalizability, and cost among costing methods, but the report doesn’t provide specific comparisons across costing methods.”**

**(4) and (5) Thank you reviewer for your keen eye. We have corrected both problems.**

This type of direct comparison of costing methodologies is of high interest to health economists, especially those who engage in primary cost data collection. The study is thoughtfully executed and the article is well-written and -organized.

6. PLOS authors have the option to publish the peer review history of their article ([what does this mean?](https://urldefense.com/v3/__https:/journals.plos.org/plosone/s/editorial-and-peer-review-process*loc-peer-review-history__;Iw!!NFcUtLLUcw!X8MNFLtBlSgg1grTEzuk3Xy5PjrJKTVfO9lp2ydgIYrm27aS1i9_aQBgXqr26HUa3hflqzgsrhExTwZaa2aEhII$)). If published, this will include your full peer review and any attached files.


If you choose “no”, your identity will remain anonymous but your review may still be made public.


**Do you want your identity to be public for this peer review?** For information about this choice, including consent withdrawal, please see our [Privacy Policy](https://urldefense.com/v3/__https:/www.plos.org/privacy-policy__;!!NFcUtLLUcw!X8MNFLtBlSgg1grTEzuk3Xy5PjrJKTVfO9lp2ydgIYrm27aS1i9_aQBgXqr26HUa3hflqzgsrhExTwZaXpnHF1Y$).

Reviewer #1: **Yes:** Katherine Robertus Vilain

[NOTE: If reviewer comments were submitted as an attachment file, they will be attached to this email and accessible via the submission site. Please log into your account, locate the manuscript record, and check for the action link "View Attachments". If this link does not appear, there are no attachment files.]

While revising your submission, please upload your figure files to the Preflight Analysis and Conversion Engine (PACE) digital diagnostic tool, [https://pacev2.apexcovantage.com/](https://urldefense.com/v3/__https:/pacev2.apexcovantage.com/__;!!NFcUtLLUcw!X8MNFLtBlSgg1grTEzuk3Xy5PjrJKTVfO9lp2ydgIYrm27aS1i9_aQBgXqr26HUa3hflqzgsrhExTwZaGUce0Fw$). PACE helps ensure that figures meet PLOS requirements. To use PACE, you must first register as a user. Registration is free. Then, login and navigate to the UPLOAD tab, where you will find detailed instructions on how to use the tool. If you encounter any issues or have any questions when using PACE, please email PLOS at [figures@plos.org](mailto:figures@plos.org). Please note that Supporting Information files do not need this step.

*In compliance with data protection regulations, you may request that we remove your personal registration details at any time.* [*(Remove my information/details)*](https://urldefense.com/v3/__https:/www.editorialmanager.com/pone/login.asp?a=r__;!!NFcUtLLUcw!X8MNFLtBlSgg1grTEzuk3Xy5PjrJKTVfO9lp2ydgIYrm27aS1i9_aQBgXqr26HUa3hflqzgsrhExTwZaqFhppDs$)*. Please contact the publication office if you have any questions.*
